# Observing Cryptocurrencies through Robust Anomaly Scores

**DOI:** 10.3390/e24111643

**Published:** 2022-11-11

**Authors:** Geumil Bae, Jang Ho Kim

**Affiliations:** 1 Industrial and Systems Engineering, Korea Advanced Institute of Science and Technology (KAIST), Daejeon 34141, Korea; 2 Department of Industrial and Management Systems Engineering, Kyung Hee University, Yongin-si 17104, Korea; 3 Department of Big Data Analytics, Graduate School, Kyung Hee University, Yongin-si 17104, Korea

**Keywords:** cryptocurrency, anomaly score, Mahalanobis distance, minimum covariance determinant, shrinkage estimators

## Abstract

The cryptocurrency market is understood as being more volatile than traditional asset classes. Therefore, modeling the volatility of cryptocurrencies is important for making investment decisions. However, large swings in the market might be normal for cryptocurrencies due to their inherent volatility. Deviations, along with correlations of asset returns, must be considered for measuring the degree of market anomaly. This paper demonstrates the use of robust Mahalanobis distances based on shrinkage estimators and minimum covariance determinant for observing anomaly scores of cryptocurrencies. Our analysis shows that anomaly scores are a critical complement to volatility measures for understanding the cryptocurrency market. The use of anomaly scores is further demonstrated through portfolio optimization and scenario analysis.

## 1. Introduction

Even though investments in cryptocurrencies were initially viewed as risky bets, increased participation by individuals as well as institutions have been transforming those views, as the cryptocurrency market is now perceived as a new asset class for many investors. With these revolutions, there have been numerous studies with the overall objective of understanding the cryptocurrency market [1,2,3] or, more specifically, cryptocurrencies as investment assets. Much effort has been put into analyzing diversification effects and evaluating cryptocurrencies as an asset class [4,5,6,7,8]. Others have focused on diversification across cryptocurrencies [9] and cross-correlation among themselves [10,11]. Predicting price movement of cryptocurrencies using social media data [12], the economic and political uncertainty of the crypto market [13,14], and liquidity of cryptocurrencies [15,16] have also been studied. Due to large swings in cryptocurrencies, analyses also focus on the volatility of the market. Many studies have investigated risk factors of cryptocurrencies [17,18,19,20] and examined models for volatility forecasting [21,22], including forecasts of daily value-at-risk [23]. 

In this article, the risk and volatility of the cryptocurrency market are further examined but from a macro view of observing anomaly scores of market movements. The analysis is based on viewing cryptocurrencies through anomaly scores measured with Mahalanobis distance and its robust variations. The analysis has two significant contributions. First, while cryptocurrencies are generally understood as being more volatile compared to traditional assets, observing anomaly scores provides a standardized framework for identifying unlikely or outlier events, where anomaly calculations incorporate mean, variance, and correlations. Furthermore, anomaly scores are analyzed through cryptocurrency returns as well as risk factors, and robust formulations are proposed to handle extreme outliers in cryptocurrencies. Second, anomaly scores can enhance portfolio management and scenario analysis of cryptocurrencies. Anomaly scores can act as an indicator of abnormal market conditions, and they can also portray a statistical picture of historical events that provide a medium for measuring historical likelihood as well as estimated likelihood of future scenarios. Performing scenario analyses using risk factors allows a more intuitive and rational interaction with the crypto market. Overall, the analysis provides a practical example of analyzing the crypto market from a more macro perspective that is a valuable complement to volatility analysis of cryptocurrencies.

## 2. Methodology

Anomaly scores of market movements are measured with Mahalanobis distance (MD), which is a multivariate extension of z-scores, and it is computed by standardizing the deviation from mean with the covariance matrix:

$$\mathrm{MD}\left(r\right)=\sqrt{{\left(r-\mu \right)}^{T}\Sigma^{-1}\left(r-\mu \right)}$$

where $\mu \in {\mathbb{R}}^{n}$ is the mean vector and $\Sigma \in {\mathbb{R}}^{n\times n}$ is the covariance matrix of a random vector $r\in {\mathbb{R}}^{n}$. While it assumes an elliptical distribution, MD has been shown to be effective in analyzing risks of financial markets [24]. Based on MD, the anomaly score is defined as:

$$A\left(r\right)=\mathrm{MD}\left(r\right)/\sqrt{n}$$

to correct for an increase in MD caused by a larger number of variables *n* for measuring distance [25].

Due to frequent spikes in cryptocurrency movements, MD becomes sensitive to the choice of investment period; mean and covariance used in the calculation of MD are highly sensitive to outliers in price movements. Therefore, in this study, robust MDs were proposed for examining cryptocurrencies. The first proposed robust approach is taken from portfolio optimization where shrinkage estimators are used for computing MD. Mean vector was estimated with the Bayes–Stein estimator [26], and covariance matrix was shrunk using Ledoit and Wolf’s [27] approach with a diagonal target. The second robust approach employed in this study is minimum covariance determinant for computing first and second moments without outliers in returns [28]. Even though cryptocurrency returns are not normally distributed [29], the robust MD methods provide a framework for comparing robust anomaly scores. In Section 4, the empirical results compare MD when mean and covariance are estimated from either the entire period (i.e., finding distance relative to the overall movement) or the most recent 104 weeks (i.e., finding distance relative to the market condition during the most recent two years, since March 2000).

More importantly, anomaly scores were initially measured with the price movements of the top cryptocurrencies, and the analysis was repeated with the risk factors of cryptocurrencies. Risk factors are especially important for managing investment portfolios because risk exposure of a portfolio can be effectively measured with underlying factors, whereas financial assets often display high cross-correlations [30,31]. Recently, Liu and Tsyvinski [20] performed a comprehensive empirical asset pricing analysis on cryptocurrencies and found that cryptocurrency returns are exposed to network factors such as number of transactions or number of wallet users, but not production factors such as electricity and computing costs. Thus, network factors were chosen in our analysis to measure anomaly scores of the cryptocurrency market. Principal component analysis is not included in our experiment because principal components that explain much of the variance in cryptocurrencies are highly correlated with the more volatile currencies since 2018, such as Dogecoin. In particular, we found that the top three principal components explained over 70% of variance, where the first principal component is highly correlated with the equally weighted return of the cryptocurrencies and the second principal component is highly correlated with Dogecoin.

## 3. Data

Two sets of data were used in our analysis: price data of cryptocurrencies and data capturing network effects in cryptocurrencies. Closing prices of cryptocurrencies were retrieved from CoinMarketCap (coinmarketcap.com) [32] and Coin Metrics (coinmetrics.io). Daily price data denominated in USD were collected from 1 January 2018 to 28 February 2022, and converted into weekly returns (weekly returns were used to mitigate any inconsistency in time for computing daily closing price). The analysis focused on the crypto market since 2018 because cryptocurrency funds reveal distinct characteristics in the post-ICO (initial coin offering) bubble period [33] and the market has generally become more mature following the ICO bubble [34]. Crypto markets starting from 2018 can be distinguished as the post-ICO bubble period [33], and ICOs, along with ICO-related events, such as regulatory bans, are observed to cause sensitivity in the market [35].

Empirical tests were performed with seven different sets of weekly returns that ended in different days of the week (i.e., the first set of weekly returns end every Monday, the second set of weekly returns end every Tuesday, and so on). We focused on the top 40 currencies with the largest market capitalization, which account for over 90% of market capitalization valued in USD of the top 500 cryptocurrencies (as of 27 February 2022). Filtering the cryptocurrencies with price data available since the beginning of 2018 results in 15 currencies: Bitcoin (BTC), Ethereum (ETH), Tether (USDT), BNB, XRP, Cardano (ADA), Dogecoin (DOGE), Litecoin (LTC), Chainlink (LINK), Tron (TRX), Bitcoin Cash (BCH), Decentraland (MANA), Stellar (XLM), Ethereum Classic (ETC), and Filecoin (FIL). Since the crypto market contains many more cryptocurrencies, we also used the CCi30 index in our analysis, which is an index of the top 30 cryptocurrencies (the CCi30 index has been used as a representative index of the crypto market for analyzing liquidity [36], herding behavior [37,38], and dynamics of cryptocurrencies [39]).

Based on the analysis in [20], four factors of network effect in cryptocurrencies were collected: number of active addresses (address), number of transactions (transaction), number of transfers (transfer), and number of unique wallets (wallet). The number of wallets was obtained from Blockchain.com for its users, and the other three factors were obtained from data for Bitcoin from Coin Metrics (coinmetrics.io). Weekly growth of these four metrics were computed to match the weekly return periods of cryptocurrencies (the descriptive statistics of the 15 cryptocurrencies and the four network factors are included in the Appendix A, and further details, such as the effect of days of the week, are presented in [20]).

## 4. Empirical Results on Anomaly Score

### 4.1. Volatility of Cryptocurrencies

**Figure 1:**
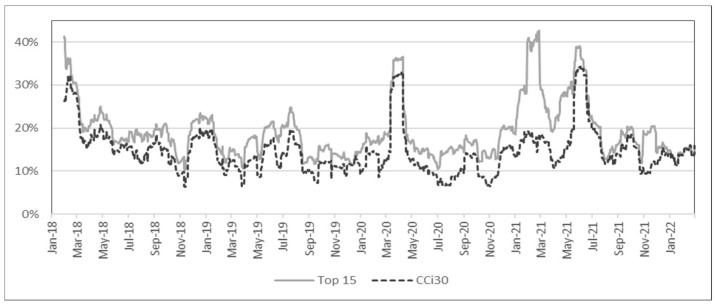
Historical annualized standard deviation (30-day rolling).

**Figure 2:**
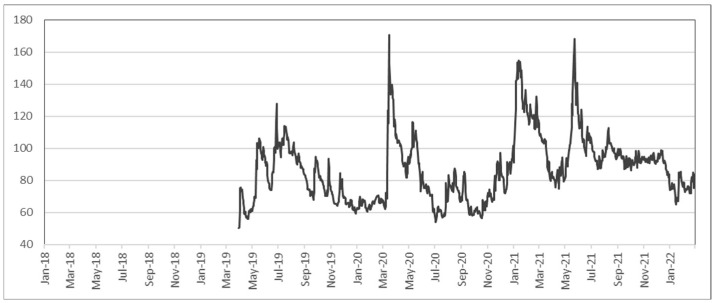
Historical values of Crypto Volatility index.

In this section, the volatility of the cryptocurrency market is observed prior to computing anomaly scores. Figure 1 shows the annualized 30-day rolling standard deviation of the top 15 cryptocurrencies and the value-weighted CCi30 index. Figure 2 shows the historical values of the Crypto Volatility index (CVI), which begins in April of 2019, but the *x*-axis has been scaled to match other figures. Anomaly score outcomes in the following sections were compared with these volatility measures to distinguish the additional value provided by anomaly scores.

### 4.2. Anomaly Scores of Cryptocurrencies

The first set of anomaly scores were computed directly from the top cryptocurrencies without the use of factors. In order to resolve sensitivity in MD, shrinkage estimators of the mean vector [26] and covariance matrix [27] were used. Shrinkage estimators combine unbiased estimators, such as sample mean, with another component with more structure. A shrinkage estimator for the vector of expected return can be expressed as:

$${\hat{\mu }}_{shrink}=\left(1-\alpha \right)\hat{\mu }+\alpha \mu_{0}1$$

where $\hat{\mu }\in {\mathbb{R}}^{n}$ is the sample mean, $\mu_{0}\in \mathbb{R}$ is the shrinkage target, $1\in {\mathbb{R}}^{n}$ is the vector of ones, and α is the shrinkage intensity. Similarly, a shrinkage estimator for the covariance matrix of returns can be written as:

$${\hat{\Sigma }}_{shrink}=\left(1-\alpha \right)S+\alpha \Sigma_{0}$$

where $S\in {\mathbb{R}}^{n\times n}$ is the sample covariance matrix and $\Sigma_{0}\in {\mathbb{R}}^{n\times n}$ is the target. In our analysis, the shrinkage target $\mu_{0}$ was set as the expected return of the portfolio with lowest risk (minimum variance portfolio) and $\Sigma_{0}$ was set as a scaled identity matrix. Even though the sample estimates can be sensitive to the estimation period, shrinking them toward a shrinkage target improves robustness [40].

**Figure 3:**
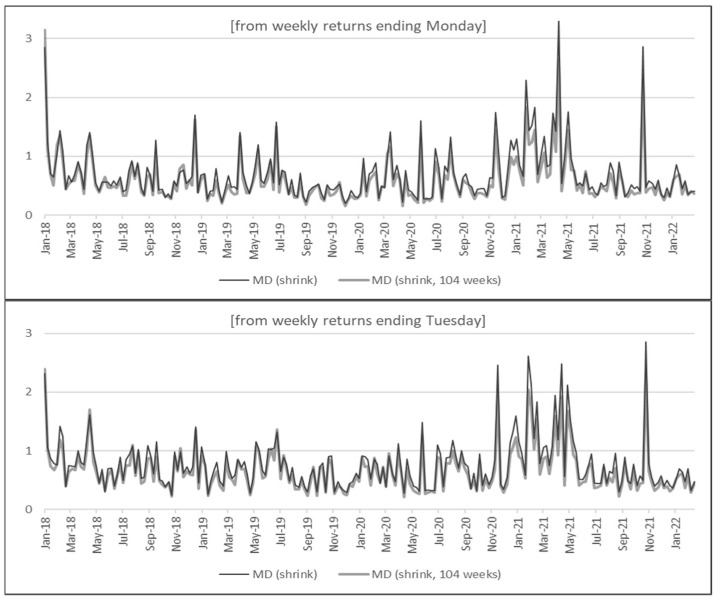

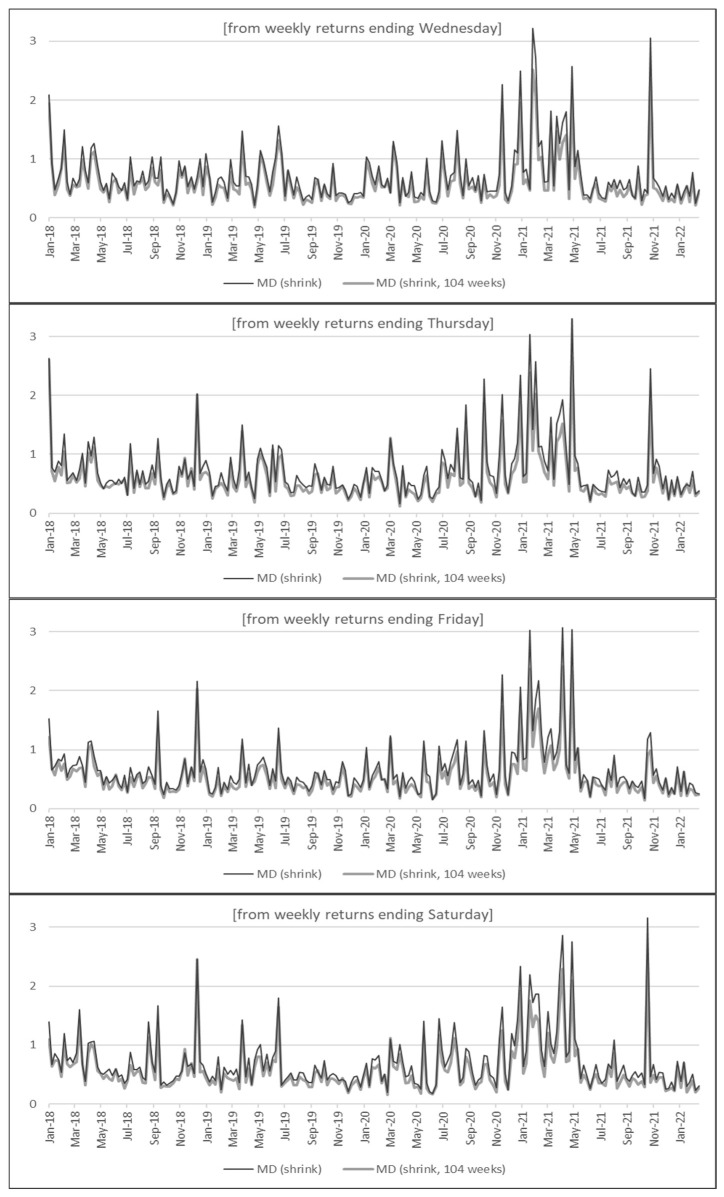

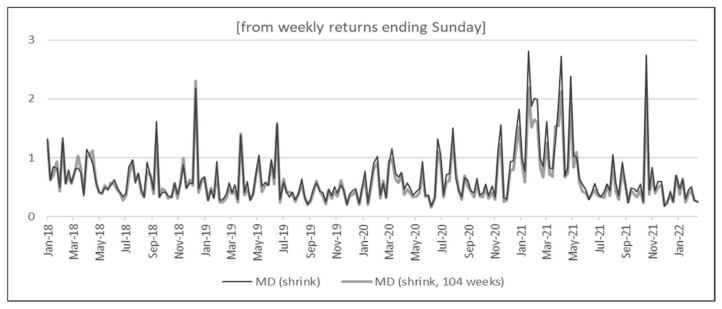
Anomaly scores from top cryptocurrencies (with shrinkage estimators).

These shrinkage estimators are frequently applied in portfolio optimization to mitigate sensitivity in the performance of optimal allocations [41,42]. Figure 3 shows anomaly scores when mean and covariance are estimated from the entire period (from January 2018 to February 2022) or from only the last 104 weeks (from March 2000 to February 2020).

Several observations are noteworthy in Figure 3. For each of the seven figures, anomaly scores are not sensitive to the estimation period, and the results are very similar between the scores based on the market condition during January 2018 to February 2022 and the condition during March 2000 to February 2022. This clearly shows the strength of using shrinkage estimators (in contrast, Figure A2 demonstrates the high sensitivity of using non-robust MD for measuring anomaly scores). Moreover, a comparison of the seven graphs in Figure 3 shows that the overall trend and spikes in anomaly scores are fairly robust to the choice of weekly return calculations. For all seven graphs, high anomaly scores are cited between late 2020 and mid-2021, followed by a short spike from around October to November of 2021. Even though there are spikes between late 2018 and mid-2019, the overall anomaly scores are relatively low from the beginning of 2018 until late 2020.

When compared with the volatility measures from Section 4.1, anomaly scores show that the high volatility periods during early-to-mid 2021 are also reflected in the anomaly scores. However, more importantly, the market movement during March to May of 2020 was rather *normal*, whereas the condition from October to November of 2021 was *abnormal*. It must be clarified that a *normal* period based on anomaly scores does not necessarily reflect a less volatile period. Since anomaly scores show squared distances from the mean that are standardized by the covariance matrix, a cryptocurrency with high volatility on average will not necessarily have a large anomaly score simply because it deviates much from the mean. This is the key reason why anomaly scores are not a substitute for market volatility but an essential complement for analyzing market movements. For example, high anomaly scores from October to November 2021 were caused by a large spike in Decentraland (MANA), which increased more than five times in less than two months. Further analysis shows that the high anomaly was not only a result of large returns but also due to changes in cross-correlation that were captured by MD. In fact, this was a period when metaverse cryptocurrencies were soaring and anomaly scores were able to capture this new wave in the market.

### 4.3. Anomaly Scores from Risk Factors

Next, anomaly scores of the cryptocurrency market were further observed using the risk factors of the crypto market. Among several studies on cryptocurrency factors, Liu and Tsyvinski [20] performed comprehensive experiments to show the significance of network factors. While network factors do not provide a complete factor model for explaining the returns and risks of cryptocurrencies, it is worth analyzing with the factors that have been identified so far as being significant.

Weekly growths of four network factors (address, transaction, transfer, and wallet) were used for computing anomaly scores, and minimum covariance determinant (MCD) was chosen for robust MD calculations. The main idea of MCD is to find a sub-sample without outliers and the sub-sample is used for computing the sample mean and covariance [43]. Shrinkage estimators are often applied when the number of variables is large, so MCD was used in our experiment for estimating robust anomaly scores when there were only a few factors [44].

**Figure 4:**
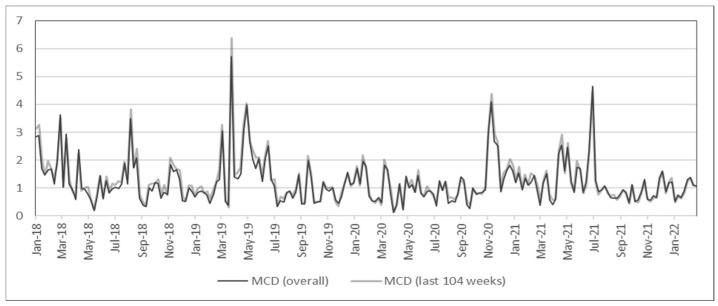
Anomaly scores from network factors (with MCD).

**Figure A1:**
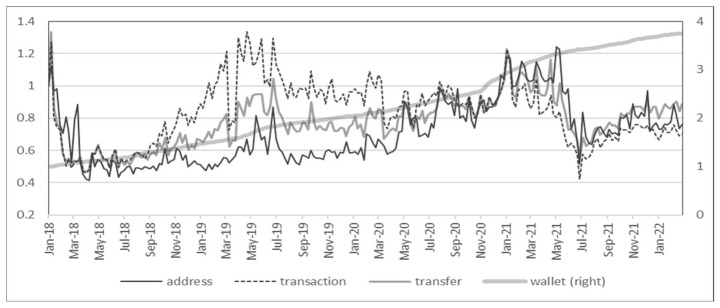
Weekly growth of the network factors (initial value: 1).

**Figure A2:**
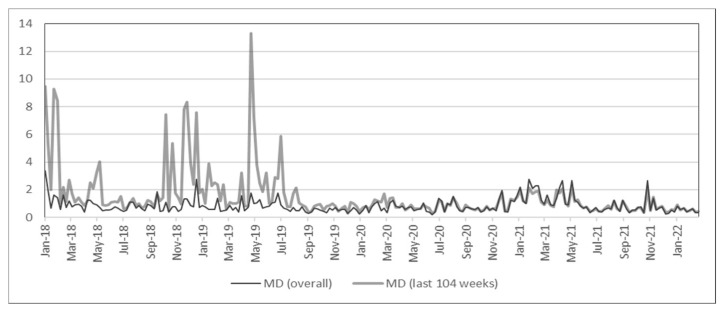
Anomaly scores from top cryptocurrencies (MD).

Here, returns were calculated for every week ending Sunday, following [20], and also because Figure 3 shows no substantial disparity among the seven graphs. In Figure 4, the anomaly scores either based on the entire period or only based on the last 104 weeks are almost identical; the robustness of MCD is also evident, similar to the robustness of shrinkage estimators in Figure 3. Additionally, the high volatility from March to May 2020 in Figure 1 and Figure 2 is not noticeable in Figure 4, which matches the anomaly results in Figure 3. While there is a large spike in March 2019 in Figure 4, this is due to a sudden decrease in the numbers of transactions and transfers (see Figure A1). Even though these factors are not able to fully describe cryptocurrency returns or risks, the main purpose of the analysis using risk factors is to demonstrate its use in scenario analysis, as demonstrated in Section 5.2.

### 4.4. Further Discussion

**Figure 5:**
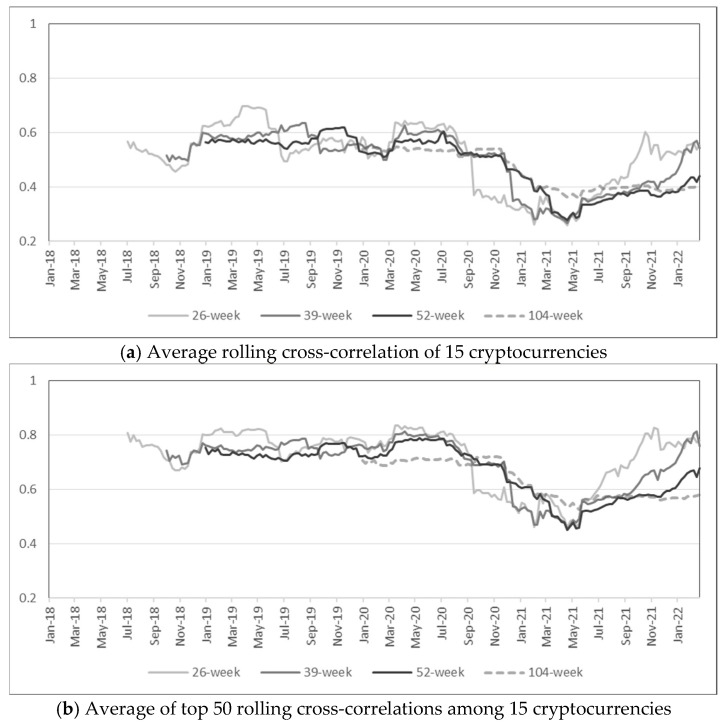
Rolling cross-correlations of weekly returns (rolling windows = 26, 39, 52, 104 weeks).

**Figure 6:**
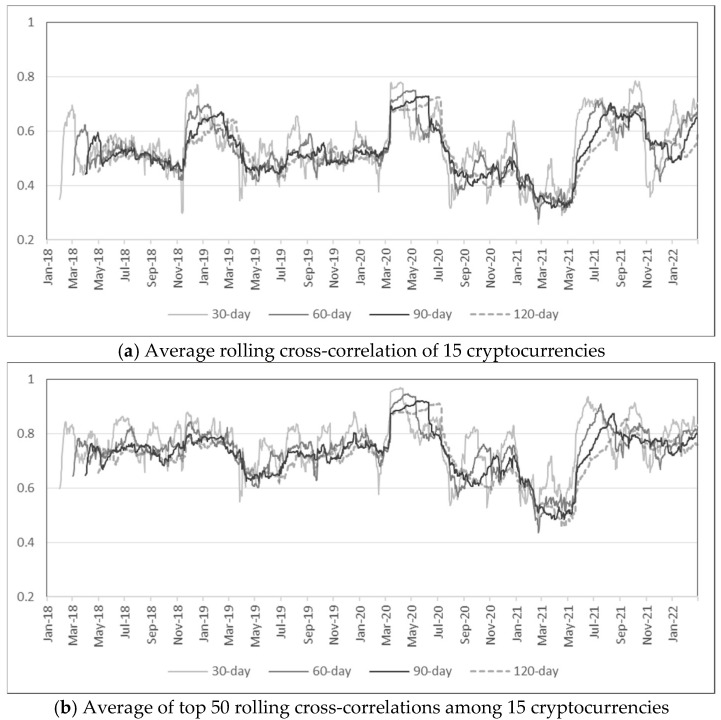
Rolling cross-correlations of daily returns (rolling windows = 30, 60, 90, 120 days).

One major distinction between measuring risk with volatility and anomaly score is that anomaly scores based on MD accounts for correlation among assets. Figure 5 plots cross-correlations among 15 cryptocurrencies for various rolling windows. The average cross-correlation is greater than 0.4 for most of the period in Panel (a), and a relatively high cross-correlation seems to be the norm due to inherent similarities among cryptocurrencies. In Panel (b), which plots the average among the top 50 cross-correlation values among 15 cryptocurrencies, the average cross-correlation is above 0.6 for most of the period. Nonetheless, there are noticeable drops in early 2021 for all the plots in Figure 5. In other words, cross-correlations among cryptocurrencies are relatively stable until late 2020 but inconsistency is observed in early 2021, which coincides with high anomaly scores. Even though average cross-correlations were more volatile when computed with daily returns as shown in Figure 6, lower cross-correlations in early 2021 are still observed, and it is especially evident from Panel (b) that the highest correlations show a significant drop in early 2021.

## 5. Portfolio Analysis Based on Anomaly Scores

As we have demonstrated so far in this study, anomaly scores provide another dimension for analyzing the risks of cryptocurrencies. Even though market anomaly provides valuable insights on its own, it can further enhance portfolio optimization and scenario analysis for investment in cryptocurrencies.

### 5.1. Incorporating Anomaly Scores into Portfolio Management

We first demonstrated how anomaly scores can be incorporated into portfolio optimization to form portfolios with lower volatility. Even when forming a diversified portfolio among cryptocurrencies, its volatility as measured by standard deviation is too high compared to traditional assets, because each cryptocurrency is volatile on its own and the correlation among cryptocurrencies are relatively high, as already discussed in Figure 5 and Figure 6. However, anomaly scores can help reduce portfolio volatility. Anomaly scores reflect abnormal market movements, so avoiding these periods reduces portfolio volatility even when forming a portfolio that only invests in cryptocurrencies.

For this backtest, rolling optimization was performed with weekly re-optimization and a lookback period of either 52 or 104 weeks. In order to focus on portfolio models with low risk, global minimum-variance (GMV) and risk-parity (equal risk contribution) models were used for optimizing portfolio weights. These are two popular models for forming an investment portfolio based on investment risk rather than expected return. The GMV portfolio model finds the optimal weights $\omega \in {\mathbb{R}}^{n}$ with the smallest risk in the mean-variance optimization framework [40,45] and is written as:

$$\underset{\omega }{\min}\;\frac{1}{2}\omega^{T}\Sigma \;\omega$$

where $\Sigma \in {\mathbb{R}}^{n\times n}$ is the covariance matrix of returns for *n* assets. The risk-parity formulation can be written as:

$$\underset{\omega }{\min}\;\overset{n}{\underset{i=1}{\sum }}\overset{n}{\underset{j=1}{\sum }}{\left(RC\left(\omega_{i}\right)-RC\left(\omega_{j}\right)\right)}^{2}\;\mathrm{where}\;RC\left(\omega_{i}\right)=\omega_{i}\frac{\partial \sigma \left(\omega \right)}{\partial \omega_{i}}$$

that minimizes discrepancies among risk contributions (*RC*) of each asset, where *RC* is measured with respect to the standard deviation σ of a portfolio [46]. The feasible portfolios were restricted to non-negative weights that sum to one, which is the most basic setting in portfolio construction [47].

On each re-optimization date, the portfolio strategy decided not to invest in cryptocurrencies (i.e., sell all positions) if the anomaly score was above a certain pre-determined limit (e.g., 1 or 2), and ex ante anomaly scores with shrinkage were computed each time from either previous 52-week or 104-week returns. A 52-week lookback period results in portfolio performance from January 2019 to February 2022, and a 104-week lookback provides performance from January 2020 to February 2022. Portfolios were constructed with no-shorting constraints, and USDT was excluded in the backtest because it had negative expected returns during this period.

Table 1 presents weekly standard deviations, annualized standard deviations, and the number of weeks over limit for several anomaly limits. The third column shows results for an equally weighted portfolio of the top 14 cryptocurrencies. The annualized volatility was above 90% without incorporating anomaly scores, but decreased to below 50% with an anomaly limit of 0.5. GMV, and risk-parity portfolios had lower standard deviation compared to the equally weighted portfolio. In particular, GMV had the lowest risk and the annualized volatility was near 40% when an anomaly limit of 0.5 was imposed. Therefore, portfolios with annualized volatility above 80% are unreasonably risky for all rational investors, which is the case without any anomaly limit, but reducing volatility to 40% may provide a viable investment option for investors with minimal risk aversion.

### 5.2. Scenario Analysis of Cryptocurrencies

A major significance of using factors for computing MD is its effectiveness in performing scenario analysis [48]. The two most important components of scenario analysis are the construction of meaningful scenarios and the probability of occurrence for the scenarios. Even though it is difficult to construct scenarios directly at the cryptocurrency level (e.g., it is challenging to form an outlook on short-term returns for a certain currency), it is more intuitive to form a logical outlook on risk factors such as the growth in total transactions or users. Furthermore, since the likelihood of a scenario is proportional to $e^{-MD/2}$, these values can be rescaled to sum to one when estimating the probability of several scenarios [48].

Here, an example is presented to demonstrate how scenarios can be formed with cryptocurrency factors when anomaly scores are computed with robust MD. Table 2 shows mean and standard deviation of weekly growth for the four factors, and the growth in weekly transactions appear to be near zero on average since the beginning of 2018. Suppose scenario analysis is performed based on the view that transactions are going to increase in the coming week; consider growth in transactions to be realized within the set {0.001%, 0.5%, 1.0%, 1.5%, …, 10.0%}. Thus, 11 scenarios are generated where transaction takes one of the 11 values, whereas the growth of the other three factors are assumed to stay unchanged (i.e., mean values from Table 2). The advantage of scenario generation from factors is clearly evident in this case. Expressing market outlook through growth in the number of transactions is intuitive even for an investor not familiar with the cryptocurrency market. More rational and detailed views can be expressed with factors.

**Figure 7:**
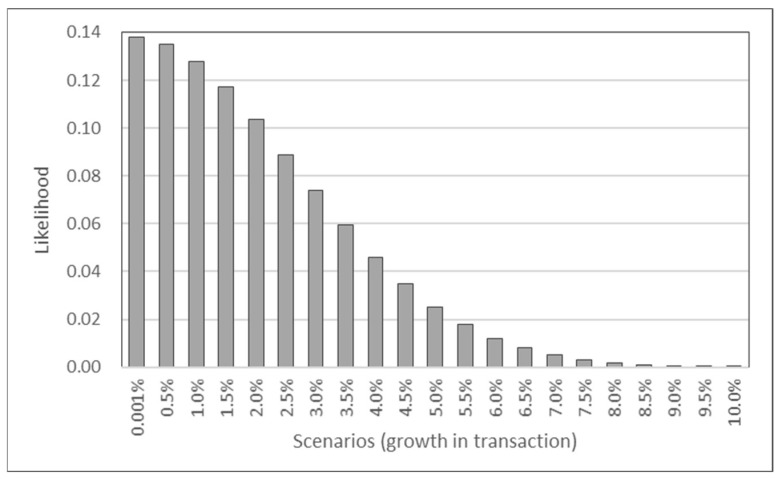
Likelihood of example scenarios on transaction.

**Figure 8:**
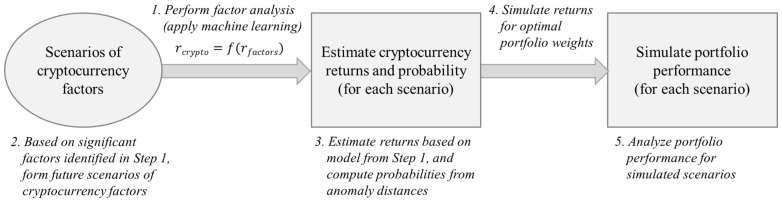
Framework for performing scenario analysis of the crypto market.

Next, anomaly scores of these scenarios provide the likelihood (probability) of occurrence for each scenario, and Figure 7 plots the likelihood for the 11 scenarios in this example. The probability of growth in weekly transaction being at least 6% is less than 5%. Thus, even though scenarios are included for cases with large transaction growth, incorporating likelihood through anomaly scores controls the influence on future outcome that are considered outliers. Finally, based on the scenario analysis of traditional assets proposed by [48], the scenarios for the crypto market can be performed as summarized in Figure 8 by applying machine learning models to identify significant factors for efficiently forming rational outlook. These scenarios can be combined with anomaly scores for simulating portfolios invested in cryptocurrencies.

## 6. Conclusions

In this article, the use of anomaly scores is illustrated for analyzing the cryptocurrency market. In addition to analyzing the volatility of the cryptocurrency market, anomaly scores of the market provide a complement to the analysis because anomalies are measured by deviation relative to variance and correlation. Specifically, robust Mahalanobis distance based on shrinkage estimators and minimum covariance determinant are shown to produce robust anomaly scores of cryptocurrencies that offer details of market anomalies that are not necessarily explained by standard volatility measures. With the use of anomaly scores as a complement to traditional volatility analyses, investment in cryptocurrencies can be further managed through a detailed understanding of normal or abnormal behavior of cryptocurrencies. Future research can be directed towards analyzing the underlying cause of the discrepancies between traditional volatility measures and the robust anomaly scores proposed in this study. One of the current shortcomings is the limited findings related to risk factors of cryptocurrencies and access to various data. Extended research into risk factors of cryptocurrencies will contribute to computing anomaly scores. Finally, further insight into abnormal behavior in cryptocurrencies will not only provide effective tools for managing investment in the crypto market but also become extremely valuable for investors expanding their assets with cryptocurrencies.

## Figures and Tables

**Table 1:** Risk performance of portfolios based on various anomaly limits.

Portfolio Model		Equally Weighted	Global Minimum-Variance		Risk-Parity	
Lookback period (weeks)		-	52	104	52	104
Without anomaly limit	std	0.128	0.112	0.114	0.125	0.137
	std (annual.) *	0.923	0.808	0.825	0.900	0.989
	number of weeks over limit	0	0	0	0	0
	total number of weeks	164	164	112	164	112
Anomaly limit = 2.0	std	0.123	0.110	0.099	0.120	0.121
	std (annual.) *	0.889	0.792	0.712	0.869	0.874
	number of weeks over limit	10	10	13	10	13
	total number of weeks	164	164	112	164	112
Anomaly limit = 1.0	std	0.104	0.089	0.088	0.101	0.107
	std (annual.) *	0.749	0.638	0.632	0.732	0.773
	number of weeks over limit	41	41	37	41	37
	total number of weeks	164	164	112	164	112
Anomaly limit = 0.5	std	0.068	0.056	0.064	0.067	0.071
	std (annual.) *	0.492	0.407	0.462	0.481	0.510
	number of weeks over limit	110	110	76	110	76
	total number of weeks	164	164	112	164	112

* Standard deviation is annualized by multiplying $\sqrt{52}$.

**Table 2:** Statistics of weekly growth (from January 2018 to February 2022).

	Mean	Std
Address	0.00764	0.1173
Transaction	0.00001	0.0896
Transfer	0.00356	0.0806
Wallet	0.00547	0.0040

## Appendix A

**Table A1:** Descriptive statistics of the top 15 cryptocurrencies (weekly returns ending Sunday from 1 January 2018 to 28 February 2022).

	BTC	ETH	USDT	BNB	XRP	ADA	DOGE	LTC	LINK	TRX	BCH	MANA	XLM	ETC	FIL
mean	0.010	0.016	0.000	0.032	0.011	0.015	0.046	0.007	0.035	0.025	0.007	0.043	0.011	0.017	0.019
std	0.105	0.143	0.006	0.191	0.193	0.175	0.376	0.148	0.216	0.292	0.196	0.299	0.185	0.210	0.211
corr	BTC	ETH	USDT	BNB	XRP	ADA	DOGE	LTC	LINK	TRX	BCH	MANA	XLM	ETC	FIL
BTC	1	0.79	0.08	0.61	0.51	0.64	0.30	0.80	0.59	0.45	0.74	0.41	0.59	0.56	0.44
ETH		1	0.10	0.62	0.53	0.71	0.32	0.78	0.63	0.53	0.76	0.45	0.67	0.67	0.44
USDT			1	−0.02	0.01	0.04	0.01	0.09	0.04	−0.12	0.18	−0.01	−0.04	0.03	0.05
BNB				1	0.45	0.58	0.24	0.60	0.51	0.63	0.50	0.41	0.54	0.51	0.38
XRP					1	0.52	0.38	0.55	0.44	0.41	0.54	0.29	0.69	0.51	0.35
ADA						1	0.34	0.64	0.52	0.46	0.65	0.35	0.72	0.57	0.39
DOGE							1	0.33	0.24	0.28	0.34	0.22	0.29	0.42	0.08
LTC								1	0.59	0.45	0.79	0.38	0.56	0.74	0.42
LINK									1	0.56	0.51	0.39	0.56	0.48	0.33
TRX										1	0.41	0.40	0.58	0.44	0.32
BCH											1	0.35	0.59	0.73	0.40
MANA												1	0.39	0.32	0.24
XLM													1	0.56	0.38
ETC														1	0.33
FIL															1

**Table A2:** Descriptive statistics of the network factors (weekly growth ending Sunday from 1 January 2018 to 28 February 2022).

	Address	Transaction	Transfer	Wallet
mean	0.006	0.004	0.004	0.006
std	0.121	0.109	0.092	0.004

## References

[1] Fang F., Ventre C., Basios M., Kanthan L., Martinez-Rego D., Wu F., Li L. (2022). Cryptocurrency trading: A comprehensive survey. Financ. Innov..

[2] Kim K., Lee M. (2021). The impact of the COVID-19 pandemic on the unpredictable dynamics of the cryptocurrency market. Entropy.

[3] Wątorek M., Drożdż S., Kwapień J., Minati L., Oświęcimka P., Stanuszek M. (2021). Multiscale characteristics of the emerging global cryptocurrency market. Phys. Rep..

[4] Chuen D.L.K., Guo L., Wang Y. (2017). Cryptocurrency: A new investment opportunity?. J. Altern. Investig..

[5] Bouri E., Molnár P., Azzi G., Roubaud D., Hagfors L.I. (2017). On the hedge and safe haven properties of Bitcoin: Is it really more than a diversifier?. Financ. Res. Lett..

[6] Bianchi D. (2020). Cryptocurrencies as an asset class? An empirical assessment. J. Altern. Investig..

[7] Sifat I. (2021). On cryptocurrencies as an independent asset class: Long-horizon and COVID-19 pandemic era decoupling from global sentiments. Financ. Res. Lett..

[8] Kim J.H. (2022). Analyzing diversification benefits of cryptocurrencies through backfill simulation. Financ. Res. Lett..

[9] Liu W. (2019). Portfolio diversification across cryptocurrencies. Financ. Res. Lett..

[10] Kwapień J., Wątorek M., Drożdż S. (2021). Cryptocurrency market consolidation in 2020–2021. Entropy.

[11] Nguyen A.P.N., Mai T.T., Bezbradica M., Crane M. (2022). The cryptocurrency market in transition before and after COVID-19: An opportunity for investors?. Entropy.

[12] Valencia F., Gómez-Espinosa A., Valdés-Aguirre B. (2019). Price movement prediction of cryptocurrencies using sentiment analysis and machine learning. Entropy.

[13] Demir E., Gozgor G., Lau C.K.M., Vigne S.A. (2018). Does economic policy uncertainty predict the Bitcoin returns? An empirical investigation. Financ. Res. Lett..

[14] Colon F., Kim C., Kim H., Kim W. (2021). The effect of political and economic uncertainty on the cryptocurrency market. Financ. Res. Lett..

[15] Leirvik T. (2021). Cryptocurrency returns and the volatility of liquidity. Financ. Res. Lett..

[16] Brauneis A., Mestel R., Theissen E. (2021). What drives the liquidity of cryptocurrencies? A long-term analysis. Financ. Res. Lett..

[17] Gregoriou A. (2019). Cryptocurrencies and asset pricing. Appl. Econ. Lett..

[18] Li J., Yi G. (2019). Toward a factor structure in crypto asset returns. J. Altern. Investig..

[19] Zhang W., Li Y., Xiong X., Wang P. (2021). Downside risk and the cross-section of cryptocurrency returns. J. Bank. Financ..

[20] Liu Y., Tsyvinski A. (2021). Risks and returns of cryptocurrency. Rev. Financ. Stud..

[21] Bouri E., Lau C.K.M., Lucey B., Roubaud D. (2019). Trading volume and the predictability of return and volatility in the cryptocurrency market. Financ. Res. Lett..

[22] Ftiti Z., Louhichi W., Ben Ameur H. (2021). Cryptocurrency volatility forecasting: What can we learn from the first wave of the COVID-19 outbreak?. Ann. Oper. Res..

[23] Pele D.T., Mazurencu-Marinescu-Pele M. (2019). Using high-frequency entropy to forecast bitcoin’s daily value at risk. Entropy.

[24] Kritzman M., Li Y. (2010). Skulls, financial turbulence, and risk management. Financ. Anal. J..

[25] Golub B., Greenberg D., Ratcliffe R. (2018). Market-driven scenarios: An approach for plausible scenario construction. J. Portf. Manag..

[26] Jorion P. (1986). Bayes-Stein estimation for portfolio analysis. J. Financ. Quant. Anal..

[27] Ledoit O., Wolf M. (2004). A well-conditioned estimator for large-dimensional covariance matrices. J. Multivar. Anal..

[28] Leys C., Klein O., Dominicy Y., Ley C. (2018). Detecting multivariate outliers: Use a robust variant of Mahalanobis distance. J. Exp. Soc. Psychol..

[29] Wątorek M., Kwapień J., Drożdż S. (2021). Financial return distributions: Past, present, and COVID-19. Entropy.

[30] Ang A. (2014). Asset Management: A Systematic Approach to Factor Investing.

[31] Madhavan A., Sobczyk A., Ang A. (2018). What’s in your benchmark? A factor analysis of major market indexes. J. Portf. Manag..

[32] Vidal-Tomás D. (2022). Which cryptocurrency data sources should scholars use?. Int. Rev. Financ. Anal..

[33] Bianchi D., Babiak M. (2022). On the performance of cryptocurrency funds. J. Bank. Financ..

[34] Vidal-Tomás D. (2021). The entry and exit dynamics of the cryptocurrency market. Res. Int. Bus. Financ..

[35] Momtaz P.P. (2020). Initial coin offerings. PLoS ONE.

[36] Manahov V. (2021). Cryptocurrency liquidity during extreme price movements: Is there a problem with virtual money?. Quant. Financ..

[37] Ajaz T., Kumar A.S. (2018). Herding in crypto-currency markets. Ann. Financ. Econ..

[38] Zhao Y., Liu N., Li W. (2022). Industry herding in crypto assets. Int. Rev. Financ. Anal..

[39] Vidal-Tomás D. (2022). The new crypto niche: NFTs, play-to-earn, and metaverse tokens. Financ. Res. Lett..

[40] Kim W.C., Kim J.H., Fabozzi F.J. (2016). Robust Equity Portfolio Management + Website: Formulations, Implementations, and Properties Using MATLAB.

[41] Disatnik D.J., Benninga S. (2007). Shrinking the covariance matrix. J. Portf. Manag..

[42] Novais R.G., Wanke P., Antunes J., Tan Y. (2022). Portfolio optimization with a mean-entropy-mutual information model. Entropy.

[43] Rousseeuw P.J. (1984). Least median of squares regression. J. Am. Stat. Assoc..

[44] Hubert M., Debruyne M. (2010). Minimum covariance determinant. Wiley Interdiscip. Rev. Comput. Stat..

[45] Markowitz H.M. (1952). Portfolio selection. J. Financ..

[46] Qian E. (2011). Risk parity and diversification. J. Investig..

[47] Kim J.H., Lee Y., Kim W.C., Fabozzi F.J. (2021). Mean–Variance Optimization for Asset Allocation. J. Portf. Manag..

[48] Czasonis M., Kritzman M., Pamir B., Turkington D. (2020). Enhanced scenario analysis. J. Portf. Manag..

